# PlasmoFAB: a benchmark to foster machine learning for *Plasmodium falciparum* protein antigen candidate prediction

**DOI:** 10.1093/bioinformatics/btad206

**Published:** 2023-06-30

**Authors:** Jonas C Ditz, Jacqueline Wistuba-Hamprecht, Timo Maier, Rolf Fendel, Nico Pfeifer, Bernhard Reuter

**Affiliations:** Methods in Medical Informatics, Department of Computer Science, University of Tübingen, 72076 Tübingen, Germany; Methods in Medical Informatics, Department of Computer Science, University of Tübingen, 72076 Tübingen, Germany; Methods in Medical Informatics, Department of Computer Science, University of Tübingen, 72076 Tübingen, Germany; Computomics GmbH, 72072 Tübingen, Germany; Institute of Tropical Medicine, University Hospital Tübingen, 72074 Tübingen, Germany; German Center for Infection Research (DZIF), Partner Site Tübingen, Tübingen, Germany; Methods in Medical Informatics, Department of Computer Science, University of Tübingen, 72076 Tübingen, Germany; Methods in Medical Informatics, Department of Computer Science, University of Tübingen, 72076 Tübingen, Germany

## Abstract

**Motivation:**

Machine learning methods can be used to support scientific discovery in healthcare-related research fields. However, these methods can only be reliably used if they can be trained on high-quality and curated datasets. Currently, no such dataset for the exploration of *Plasmodium falciparum* protein antigen candidates exists. The parasite *P.falciparum* causes the infectious disease malaria. Thus, identifying potential antigens is of utmost importance for the development of antimalarial drugs and vaccines. Since exploring antigen candidates experimentally is an expensive and time-consuming process, applying machine learning methods to support this process has the potential to accelerate the development of drugs and vaccines, which are needed for fighting and controlling malaria.

**Results:**

We developed *PlasmoFAB*, a curated benchmark that can be used to train machine learning methods for the exploration of *P.falciparum* protein antigen candidates. We combined an extensive literature search with domain expertise to create high-quality labels for *P.falciparum* specific proteins that distinguish between antigen candidates and intracellular proteins. Additionally, we used our benchmark to compare different well-known prediction models and available protein localization prediction services on the task of identifying protein antigen candidates. We show that available general-purpose services are unable to provide sufficient performance on identifying protein antigen candidates and are outperformed by our models that were trained on this tailored data.

**Availability and implementation:**

*PlasmoFAB* is publicly available on Zenodo with DOI 10.5281/zenodo.7433087. Furthermore, all scripts that were used in the creation of *PlasmoFAB* and the training and evaluation of machine learning models are open source and publicly available on GitHub here: https://github.com/msmdev/PlasmoFAB.

## 1 Introduction

Malaria is a major health problem worldwide, causing more than 247 million cases and ∼619 000 deaths in 2021 (World Health Organization et al. 2022). Almost all malaria cases are caused by *Plasmodium falciparum*, predominantly in Africa. Children, pregnant women, and malaria-naïve subjects are at high risk to develop severe malaria (Riley and Stewart 2013; Wu 2019). Furthermore, the increase in resistance to both insecticides that target the mosquito vector and anti-malaria drugs, as well as the COVID-19 pandemic, led to an increase of morbidity in several highly endemic countries in the past years (World Health Organization et al. 2021). Vaccines are very effective means in protecting against infectious diseases as recently demonstrated in the case of COVID-19. The RTS, S vaccine is the first malaria vaccine recommended by the World Health Organization (WHO) for widespread use in children in endemic settings with a substantial reduction of severe malaria cases, but limited reduction of transmission of malaria (Olotu et al. 2013; RTS,S Clinical Trials Partnership 2015). Besides this first success in fighting severe malaria, there is still an urgent need to develop an effective malaria vaccine that confers sterile protection and reduces malaria transmission. However, developing an effective malaria vaccine is still challenging due to the complex, multi-stage life-cycle of *P.falciparum*, which is genetically highly diverse and employs several immune evasion strategies. As a result, our understanding of immune responses to *P.falciparum*-specific antigens that mediate naturally acquired or experimentally induced protection is incomplete.

More than 5300 genes are expressed during the life-cycle of *P.falciparum* (Obiero et al. 2019). However, only a small subset of proteins that are expressed by *P.falciparum* is considered in current target candidate screening processes for an effective malaria vaccine (Mordmüller et al. 2017; Jagannathan and Kakuru 2022). Since most of the unused proteins have unknown function and experimental validation remains costly and time-intensive, computational methods can be used for pre-screening of proteins of interest. For example, transmembrane topology prediction is an established task in bioinformatics, where the aim is to predict how and if a protein resides in the cell membrane, i.e. predict the location and length of transmembrane domains. The class of membrane proteins is one of the most important classes of proteins for medical use. About 25–30% of natural proteins reside in the cell membrane and are, thus, often bound by antibodies during an immune reaction (Baker 2010). Another class of relevant proteins for vaccine and drug development is the class of exported proteins. Many of these fulfil important functions for parasite survival. For example, certain proteins ensure that infected red blood cells stick to the microvasculature, one of the factors that makes malaria a potentially fatal disease (Tuteja 2007; Wahlgren et al. 2017). In recent years, several scholars developed general-purpose models for sub-cellular localization prediction and offered them as prediction services to be used by the academic community (Krogh et al. 2001; Käll et al. 2007; Almagro Armenteros et al. 2017; Hallgren et al. 2022; Thumuluri et al. 2022). While general-purpose models provide researchers with an easy-to-use solution for performing prediction tasks, the lack of out-of-distribution generalization capabilities of most general-purpose models leads to sub-optimal prediction performances on novel datasets and misleading pre-screening results (Ye et al. 2021). However, training supervised machine learning models for protein antigen candidate prediction needs a sufficient amount of protein sequences with high-quality labels. Currently, only a small fraction of publicly available *P.falciparum* protein sequences have high-quality labels, making the training of models for identification of such antigens for vaccine and drug development exponentially harder. With this work, we introduce the ***Plasmo****dium* ***F****alciparum-specific* ***A****ntigen candidate* ***B****enchmark (PlasmoFAB)*, a manually pre-processed and curated dataset containing labelled protein sequences for *P.falciparum* protein antigen candidate prediction.

This article is structured as follows. We describe in detail the process of creating *PlasmoFAB* including the used data sources, pre-processing, and validation steps. Afterwards we present our experiments for predicting *P.falciparum* protein antigen candidates. Here we show the limitations of using established tools and present approaches that provide solutions to overcome these limitations. We conclude our work with a discussion about necessary actions that have to be taken in order to further improve *PlasmoFAB* and, hence, further foster the development of vaccines and drugs to control malaria.

## 2 *PlasmoFAB*: *Plasmodium falciparum*-specific protein antigen candidate benchmark

The term supervised machine learning (SL or supervised ML) summarizes techniques that correlate patterns within datasets to desired output variables, i.e. labels for classification or continuous values for regression. The foundation of using supervised ML methods for scientific discovery in medical research is curated datasets with validated and biologically meaningful output variables. Currently, there is no benchmark that fulfils this prerequisite for the exploration of *P.falciparum* protein antigen candidates. With this article, we tackle this fundamental obstacle for supporting *P.falciparum* protein antigen candidate exploration with supervised ML techniques.

**Figure 1.**
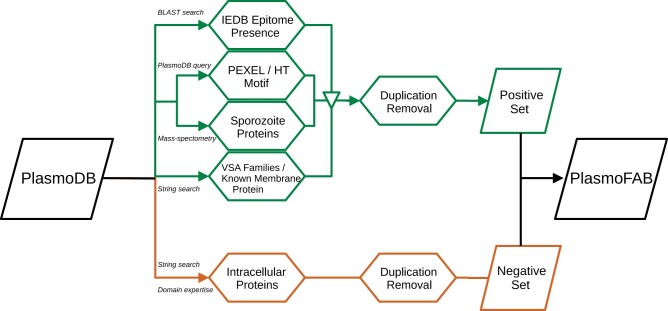
Schematic overview of the pre-processing steps for the creation of *PlasmoFAB*. The upper part of the workflow shows the pre-processing of the positive set, i.e. *Plasmodium falciparum* proteins that are either extracellular or membrane-located which renders them eligible to be considered as antigen candidates. We used knowledge-driven techniques like algorithmic homology search, mass-spectrometry, string search, and validation by published literature to create sets of proteins containing antigen candidates. These sets were merged and duplicates were removed to create the positive set. The lower part of the workflow shows the pre-processing of the negative set. We combined enzymes validated by published literature with proteins that were assigned to be intracellular by a domain expert and UniProtKB/SwissProt (reviewed) to create the negative set.

In the humoral immune response, the production of antibodies is an important step in getting rid of pathogens. To enable this response chain, pathogen-specific antigens activate B cells and their differentiation into antibody secreting plasma cells. Therefore, an antigen candidate has to be visible by the humoral immune system of the host. *Plasmodium falciparum* protein antigen candidates can be considered visible, if they are present on the outside of infected host cells, like surface proteins, transmembrane proteins, membrane-located proteins, or exported proteins. The VEuPathDB database *PlasmoDB* (Amos et al. 2022) contains the complete genome of different Plasmodium species. The protein sequences of the reference strain 3D7 of *P.falciparum*, available in *PlasmoDB*, are the data source for our curated benchmark. We only selected sequences with experimental evidence, i.e. the corresponding *P.falciparum* protein has to be referenced in published work with a unique publication identifier. However, these sequences do not have a sub-cellular location label. We combined an extensive literature search with domain expertise to create high-quality sub-cellular location labels that can be used to train ML models on the task of protein antigen candidate prediction for *P.falciparum*. In other words, *PlasmoFAB*’s positive set contains *P.falciparum* proteins that are accessible at the surface or the exterior of infected cells, like surface proteins, transmembrane proteins, membrane-located proteins, or exported proteins. On the other hand, *PlasmoFAB*’s negative set contains intracellular proteins, which are needed by the parasite to maintain the intracellular life cycle in hepatocytes or erythrocytes. The executed pre-processing steps for the creation of *PlasmoFAB* are detailed in the following section. A schematic overview of our pre-processing can be found in Fig. 1 and the basic statistics of *PlasmoFAB* are shown in Table 1.

**Table 1.** Composition of the *PlasmoFAB* benchmark.^a^

Positive set (unique total = 438)			Negative set (unique total = 384)		
Inclusion criterion	Identified by	# proteins	Inclusion criterion	Identified by	# proteins
IEDB epitope	BLAST match (high confidence)	57	Intracellular proteins	Combined string and literature search; domain expertise	384
IEDB epitope	BLAST match (medium confidence)	60			
PEXEL/HT motif	*PlasmoDB* query	265			
Sporozoite proteins	Mass-spectrometry (Swearingen et al. 2016)	13			
VSA family/membrane proteins	Combined string and literature search	302			

a The difference between the sum of sequences in each inclusion criterion and the total number of unique sequences in the positive set occurs due to the fact that some proteins fulfil more than one inclusion criterion. These proteins were not duplicated, resulting in the mismatch between the sum of proteins in each criterion and the total number of proteins in *PlasmoFAB*.

### 2.1 IEDB epitopes

An epitope is the part of an antigen that is recognized by the immune system of a host organism, i.e. the binding site of an antibody. The Immune Epitope Database (IEDB, https://www.iedb.org/, Vita et al. 2019) contains sequences of known epitopes. We used exact string matching and BLAST similarity matching to compare *P.falciparum* protein sequences with sequences contained in the IEDB. Proteins that either contained exact matches of epitope sequences or a positive BLAST hit with high or medium confidence score were labelled as antigen candidates for our benchmark.

### 2.2 PEXEL/HT motif

The majority of *P.falciparum* proteins that are either exported into the extracellular space by the parasite or integrated into the membrane of infected erythrocytes contain a specific amino acid sequence called Plasmodium exported element (PEXEL) or host targeting (HT) (Osborne et al. 2010; Jonsdottir et al. 2021). Therefore, the presence of this motif is a strong indicator of a protein antigen candidate. *PlasmoDB* indicates the presence of the PEXEL/HT motif within a sequence by a flag in one of its data fields. For *PlasmoFAB*, we included all proteins with the PEXEL/HT motif as positive antigen candidates.

### 2.3 VSA families and known membrane proteins

Variant surface antigen (VSA) families describe proteins that are typically located on cell surfaces. There are three known VSA families in the *P.falciparum* genome: *P.falciparum* erythrocyte membrane protein 1 (PfEMP1), repetitive interspersed family (RIFIN), and sub-telomeric variable open reading frame (STEVOR). The first family, PfEMP1, summarizes proteins that are expressed on the surface of infected erythrocytes during the trophozoite and schizont stage of the infection cycle. These proteins are mainly responsible for effective evasion of immune responses (Wahlgren et al. 2017). Proteins belonging to the RIFIN family are exported onto the cell surface of infected erythrocytes as well. They mediate the sequestration of erythrocytes which results in erythrocyte rosetting that further helps parasites to evade immune responses and can block the blood flow (Wahlgren et al. 2017). Similar to the other two VSA families, STEVOR proteins are also used by *P.falciparum* parasites to evade host immune responses. They play active roles in the trophozoite, schizont, merozoite, and gametocyte stages of the infection cycle (Gardner et al. 2002; Wahlgren et al. 2017). Beside the members of VSA families, there are a number of known membrane proteins. In the sporozoite stage, those include thrombospondin-related anonymous protein (TRAP), also known as sporozoite surface protein 2 (SSP2), apical membrane antigen 1 (AMA1), liver stage antigen 1 (LSA1), and exported protein 1 (Exp-1), also known as circumsporozoite-related antigen (CRA). Additionally, we included known surface proteins that can be found in other stages of the infection cycle like the family of monomeric serine-threonine protein kinases (FIKK, Anil Kumar et al. 2021), the helical intersperse sub-telomeric family of exported proteins (PHIST, Tarr et al. 2014), and the multigene family of cytoadherence linked asexual gene (CLAG, Gupta et al. 2015).

Each entry in *PlasmoDB* has a textual product description field containing information about the sample in textual form. We performed a string search on the textual product description field using the names of the VSA families as search terms: ‘*PfEMP1*’, ‘*RIFIN*’, ‘*STEVOR*’. For additional known membrane and exported proteins, we did not only included the names but also descriptive search terms since the textual product description field is not standardized. The additional search terms were ‘*surface*’, ‘*circumsporozoite*’, ‘*membrane*’, ‘*exported*’, ‘*serine repeat antigen*’, ‘*TRAP*’, ‘*FIKK*’, ‘*GLURP*’, ‘*CLAG*’, ‘*PHIST*’, and ‘*GPI-anchor*’. However, the source and rationale behind the annotation in *PlasmoDB*’s textual product description field are not always disclosed. To ensure that only validated membrane and exported proteins are included in our benchmark, we performed a literature search for each protein that was selected by our string search and included only proteins with published experimental evidence into our benchmark. To further enrich the set of known membrane proteins, we added a list of sequences validated by the UniProtKB/SwissProt (reviewed) database. This database contains high quality, manually annotated proteins sequences (The UniProt Consortium 2023).

### 2.4 Sporozoite surface-exposed proteins

The authors in Swearingen et al. (2016) used mass-spectrometry to identify potential surface-exposed sporozoite proteins of *P.falciparum*. They assigned priority scores to each investigated protein ranging from 1 (high confidence) to 6 (low confidence). We downloaded the publicly available data from Swearingen et al. (2016) and selected all proteins with a priority score from 1 to 3. We used the unique transcript ID of these proteins to merge this information into the *PlasmoDB* data table and included them into our benchmark as antigen candidates.

### 2.5 Intracellular proteins

The pre-processing steps described above added positive samples, i.e. *P.falciparum* protein antigen candidates, to our benchmark. However, *PlasmoFAB* needs negative samples, i.e. proteins that are not *P.falciparum* protein antigen candidates, to be usable for training of supervised ML methods. A model can only learn to detect true protein antigen candiates, if a set of high-quality negative samples, a so-called negative set, is available. Similar to the positive samples, we curated the negative samples to ensure that only intracellular *P.falciparum* proteins are included into the negative set. Intracellular proteins can only leave the cytoplasm in specific situations that do not reliably occur in the infection cycle, like the burst of an infected erythrocyte or if macrophages digest an infected erythrocyte and subsequently present an intracellular protein as an antigen. However, due to the unreliability of these incidents and the fact that both can only occur late in the infection cycle, intracellular proteins are not suitable as antibody targets. Enzymes constitute a subset of intracellular proteins. We performed a string search with the term ‘*ase’ on *PlasmoDB*’s textual product description field and included all proteins with published experimental evidence of being enzymes into the negative set of our benchmark. While there is a small number of enzymes that are exported to the cell membrane, we made sure to exclude all enzymes from the negative set for which published experimental evidence of being membrane-located exists. Furthermore, we included a list of known intracellular proteins compiled by a domain expert and a list of intracellular proteins validated by UniProtKB/SwissProt (reviewed).

## 3 Utilizing machine learning for *Plasmodium falciparum* protein antigen candidate exploration

Manually exploring *P.falciparum* proteins for potential antigen candidates is a time-consuming and expensive procedure. With the help of our curated benchmark, we can utilize supervised ML to accelerate the process with a pre-screening of potential proteins that reduces the required workload of researchers in the laboratory. The usefulness of such a pre-screening process highly depends on the accuracy that prediction models are able to achieve. We compared the performance of several ML approaches that are commonly used for textual data, especially for biological sequences. The used methods include a kernelized support vector machine (SVM) utilizing the oligo kernel (Meinicke et al. 2004), the protein language model embedding ESM-1b (Rives et al. 2021) combined with a logistic regression (LR) classifier as well as an SVM, and the protein language model embedding ProtT5 (Elnaggar et al. 2020), which we also combined with an LR classifier and an SVM. Furthermore, we also tested the performance of existing protein localization prediction tools on the *P.falciparum* protein antigen candidate prediction task. These tools are publicly offered as a service for protein localization prediction tasks and included TMHMM (Krogh et al. 2001), DeepTMHMM (Hallgren et al. 2022), DeepLoc 1.0 (Almagro Armenteros et al. 2017), DeepLoc 2.0 (Thumuluri et al. 2022), and Phobius (Käll et al. 2007). To ensure a fair comparison between pre-trained prediction services and our self-trained models, we defined a test set that was separated from the training data before model training was performed. We used *MMseqs2* (Zimmermann et al. 2018; Gabler et al. 2020) to ensure that each sequence in the test set had at most 30% homology to sequences in the training set, which is the default setting of *MMseqs2*. The test set consists of 60 sequences (30 antigen targets and 30 intracellular proteins) with the remaining 788 sequences in *PlasmoFAB* used as a training set. All performance measures shown in this section are computed on the test set.

To assess the performance of each method, we used three performance measures that are widely used in computational biology due to their ability to handle imbalanced data with relative ease. First, we used balanced accuracy, which has different definitions in literature. We used the arithmetic mean of sensitivity and specificity (Pedregosa et al. 2011) given by where TP is the number of correctly predicted protein antigen candidates (i.e. true positives), FP is the number of wrongly predicted protein antigen candidates (i.e. false positives), TN is the number of correctly predicted intracellular proteins (i.e. true negatives), and FN is the number of wrongly predicted intracellular proteins (i.e. false negatives). Additionally, we used the F_1_-score that is the harmonic mean of precision and recall (Taha and Hanbury 2015) given by with TP, FP, and FN defined in the same way as above. Finally, we also included the Matthews correlation coefficient (MCC, Chicco and Jurman 2020), which is widely recognized as one of the most reliable performance measures for binary classification on biological data. The MCC is defined as


(1) $${\text{Acc}}_{\text{bal}}=\frac{1}{2}\left(\frac{\text{TP}}{\text{TP}+\text{FN}}+\frac{\text{TN}}{\text{TN}+\text{FP}}\right),$$

(2) $$F_{1}=\frac{2\text{TP}}{2\text{TP}+\text{FP}+\text{FN}},$$

(3) $$\text{MCC}=\frac{\text{TP}\cdot \text{TN}-\text{FP}\cdot \text{FN}}{\sqrt{\left(\text{TP}+\text{FP})(\text{TP}+\text{FN})(\text{TN}+\text{FP})(\text{TN}+\text{FN}\right)}}.$$

Again, the definition of TP, FP, TN, and FN are the same as above. Since the classes in *PlasmoFAB* are balanced, we also report precision, recall, and specificity to provide a quick overview over the distribution of FN and FP for the predictions of the tested models.

### 3.1 Using *PlasmoFAB*’s training sequences for model training

Hyperparameter optimization and model selection was exclusively performed on *PlasmoFAB*’s training sequences to avoid information leakage from the test sequences. As a baseline model, we trained a kernelized SVM utilizing the oligo kernel, a kernel function that was specifically developed for biological sequences (Meinicke et al. 2004). This kernel computes the similarity of two sequences based on *k*-mer occurrence with a tunable degree of positional uncertainty. The SVM that was trained for *P.falciparum* protein antigen candidate prediction had three hyperparameters that needed to be optimized: the *k*-mer length, the positional uncertainty parameter σ, and the regularization parameter $C_{\text{SVM}}$. We performed a grid search utilizing repeated nested cross-validation to optimize all three hyperparameters. The resulting choices were k=1, σ=18, and $C_{\text{SVM}}=0.001$.

Additionally, we used two more complex language embedding models that are commonly used for biological sequences: ESM-1b and ProtT5. The first, ESM-1b, is a pre-trained transformer model (Rives et al. 2021), which is offered as a feature generator for downstream prediction models. It was developed to be used on biological sequences. ESM-1b follows the self-supervised bidirectional encoder representation from transformation (BERT) pre-training procedure. This language model is a transformer architecture with 33 layers and utilizes self-attention with 20 attention heads. The resulting features have a dimensionality of 1280 with a token context size of 1024. ESM-1b was trained on sequence clusters derived from the UniProt database (Bairoch et al. 2005). We refer the interested reader to the original publication for all technical details about ESM-1b. The token context size together with a positional encoding of fixed length limits input sequences to a maximum of 1024 characters. Since there is a significant number of sequences in *PlasmoFAB* that exceed this character limit, we followed published recommendations to cut the middle part of sequences that exceed the 1024 character limitation (Thumuluri et al. 2022) to be able to use ESM-1b on our benchmark. In total, 261 sequences were affected by this cutting procedure. The computed feature embeddings were used as inputs for the two tested downstream prediction models, LR and SVM. Again, we exclusively optimized the regularization parameters $C_{\text{LR}}$ and $C_{\text{SVM}}$, respectively. After performing the grid search, the optimal parameter choices were $C_{\text{LR}}=0.15$ and $C_{\text{SVM}}=20$.

The second language embedding that we used was ProtT5-XL-UniRef50 (ProtT5, Elnaggar et al. 2020). This transformer model, based on the language model T5 (Raffel et al. 2020), is specifically developed for biological data and prediction tasks. Similar to ESM-1b, ProtT5 acts as a feature generator for downstream prediction models. In contrast to other language models, ProtT5 follows an encoder–decoder approach and uses a simplified BERT training objective. The architecture employs 24 layers and also utilizes self-attention with 32 attention heads. ProtT5 has an embedding dimensionality of 1024. Since ProtT5 does not use a positional encoding of fixed length but learns a positional encoding for each attention head, the length of input sequences is not limited in theory. ProtT5 was first pre-trained on the BFD database (Steinegger et al. 2019) and fine-tuned on UniRef50 (Suzek et al. 2015). We refer the interested reader to the original publication for all technical details about ProtT5. Although sequence length is not limited when using ProtT5, finite computation power limits the usable sequence length in practice. With the computing resources available to us, an Nvidia Tesla V100 with 32GB RAM, the maximal usable sequence length was 6000 residues. Longer sequences were shortened in the same way we shortened sequences for ESM-1b. Five sequences in *PlasmoFAB* were affected by this reduction of sequence length. Again, we used the feature embedding as inputs for the two downstream prediction models, LR and SVM, and optimized the regularization parameter via a grid search. The optimal parameters were $C_{\text{LR}}=0.2$ and $C_{\text{SVM}}=2.0$.

### 3.2 Evaluating prediction models on *PlasmoFAB*’s test sequences

Table 2 shows the performance of all models on *PlasmoFAB*’s test set. The models trained by ourselves can be directly applied to the test set. Since the publicly available prediction services do not always provide a binary output, we converted the prediction output for each service into a binary label. TMHMM and Phobius provide topology predictions for input sequences and we assigned a positive label to all samples with at least one predicted transmembrane helix or at least one predicted extracellular region. Otherwise the sample was assigned a negative label. DeepTMHMM refines the prediction of TMHMM by providing a label for each residue in an input sample. For the DeepTMHMM output, we assigned a positive label to all samples with residues that had the membrane domain label (‘*M*’) assigned. Furthermore, a positive label was assigned to samples where DeepTMHMM predicted the outside cell label (‘*O*’) for all residues. If none of these conditions was fulfilled, the sample was assigned a negative label. DeepLoc 1.0 and 2.0 are tools for subcellular localization prediction and, hence, offer a multi-label output. Each label corresponds to a different subcellular localization. We used the top predicted label for each input sample. If this label was ‘cell membrane’ or ‘extracellular’, the sample was assigned a positive label, otherwise a negative label was assigned.

**Table 2.** Performance of trained prediction models and prediction services on *PlasmoFAB*’s test set.^a^

Model	MCC	F1	Bal. Acc.	Precision	Recall	Specificity
${\text{SVM}}_{\text{oligo}}$	0.3145	0.5882	0.6500	0.7143	0.5000	0.8000
${\text{LR}}_{\text{ESM}1b}$	0.7071	0.8000	0.8333	**1.0000**	0.6667	**1.0000**
${\text{SVM}}_{\text{ESM}1b}$	0.7071	0.8000	0.8333	**1.0000**	0.6667	**1.0000**
${\text{LR}}_{\text{ProtT}5}$	**0.7338**	**0.8235**	**0.8500**	**1.0000**	0.7000	**1.0000**
${\text{SVM}}_{\text{ProtT}5}$	0.6917	0.8077	0.8333	0.9545	0.7000	0.9666
DeepTMHMM	0.4395	0.6909	0.7167	0.7600	0.6333	0.8001
DeepLoc 2.0	0.4009	0.7079	0.7009	0.6000	0.6923	0.7095
DeepLoc 1.0	0.2691	0.6071	0.6357	0.5667	0.6538	0.6176
TMHMM	0.3015	0.6316	0.6500	0.6667	0.6000	0.7000
Phobius	0.2722	0.6667	0.6333	0.6111	**0.7333**	0.5333

a We trained different models on *PlasmoFAB*’s training set including a support vector machine utilizing the oligo kernel (${\text{SVM}}_{\text{oligo}}$), a combination of the a linear regression with either ESM1b or ProtT5 language model embedding (${\text{LR}}_{\text{ESM}1b}$ and ${\text{LR}}_{\text{ProtT}5}$), and a support vector machine combined with either ESM1b or ProtT5 language model embedding (${\text{SVM}}_{\text{ESM}1b}$ and ${\text{SVM}}_{\text{ProtT}5}$). Furthermore, we used publicly available, pre-trained prediction services on *PlasmoFAB*’s test set. These services include Phobius, TMHMM, DeepTMHMM, Deeploc 1.0, and Deeploc 2.0. The highest numbers are indicated by boldface values.

Our results show that models directly trained on *PlasmoFAB* training set clearly outperform the available prediction services. The best performance was achieved by combining ProtT5 feature embedding with LR. None of the tested prediction services was able to achieve a comparable performance to the specialized models.

## 4 Discussion

Computational antigen pre-screening with machine learning methods can drastically reduce time- and resource-consuming experimental exploration procedures and, thereby, accelerate development of drugs and vaccines. However, these computational pre-screening methods heavily depend on high-quality data to produce reliable results. In this work, we take important steps towards utilizing computational pre-screening for Malaria drug and vaccine development by providing *PlasmoFAB*, a benchmark that consists of *P.falciparum*-specific protein sequences with curated labels that distinguish between protein antigen candidates and intracellular proteins.

Experimental validation is the gold standard to determine subcellular localization labels for proteins. We ensured that each label in PlasmoFAB achieves this gold standard or, if experimental validation is not feasible, comes as close to the gold standard as possible. As detailed in Section 2, the biggest subgroup of proteins that were assigned as antigen candidates was the group of VSA family members and known membrane proteins. We performed an exhaustive literature search and only included proteins into this subgroup for which published experimental evidence exists. Other subsets with experimentally validated labels are sporozoite proteins and proteins that contain the PEXEL/HT motif. Sporozoite proteins were validated by mass-spectrometry (Swearingen et al. 2016). PEXEL/HT motif occurrence is a property of the protein sequence. This property is experimentally validated since *PlasmoFAB* only includes experimentally validated protein sequences. Furthermore, there is experimental evidence that *P.falciparum* parasites use the PEXEL/HT motif to export proteins (Osborne et al. 2010; Jonsdottir et al. 2021). This supports our decision to include PEXEL/HT motif occurrence as an indication of protein antigen candidates. The last remaining subgroup in *PlasmoFAB*’s positive set are proteins with known epitopes. IEDB only includes epitopes that are experimentally validated and we used BLAST to perform similarity matching between IEDB entries and *P.falciparum* protein sequences. Although BLAST does not fulfil the gold standard of experimental validation, it is widely considered as the gold standard for sequence similarity matching. By restricting ourselves to BLAST matches with high or medium confidence, we ensured that the reduction in label quality of proteins in this subgroup is minimized. *PlasmoFAB*’s negative set contains two groups of proteins: enzymes and intracellular proteins. We performed an exhaustive literature search to ensure that all included enzymes have experimental evidence of being intracellular. We excluded enzymes, if there is at least one publication with experimental evidence that suggests that the enzyme is being exported outside the cell. The other subgroup, intracellular proteins, were classified by a domain expert. While this does not fulfil the gold standard of experimental validation, we ensured to minimize the reduction in label quality by using domain expertise.

*PlasmoFAB* uses data that belongs to the *P.falciparum* strain 3D7. The genome of this specific strain of the *P.falciparum* parasite was the first to be published by Gardner et al. (2002). It is still today one of the most important information sources for malaria research (Olotu et al. 2013; RTS,S Clinical Trials Partnership 2015; Mordmüller et al. 2017; Jagannathan and Kakuru 2022). Therefore, we made the decision to concentrate on *P.falciparum* strain 3D7 for the first version of *PlasmoFAB*. For future work, we want to further refine *PlasmoFAB* by deriving high-quality labels for protein sequences of other *P.falciparum* strains in order to incorporate as much information about *P.falciparum* protein antigen candidates as possible into our benchmark.

One potentially surprising result is the sub-optimal performance of publicly available prediction services, like DeepTMHMM or DeepLoc 2.0, even though these services are relatively new and show impressive performance capabilities in their respective manuscripts. Our results do not provide evidence that the published performance capabilities of these models are overly optimistic or that they should not be used in general. On the contrary, we would like to emphasize that prediction services provide a fast and easy-to-use way for researchers without a strong background in machine learning to utilize prediction models in their research or the possibility to use prediction models even if not enough data for model training is available. However, our results highlight one common problem of general purpose models: their lack of out-of-distribution generalization (Ye et al. 2021). Models learn certain aspects of the training data’s distribution and allow trained models to achieve high prediction performance of unseen data as long as these data points came from the same distribution. However, if those unseen data points came from a different distribution, there is no guarantee that the model will be able to reliably make predictions on the new data. We see this out-of-distribution generalization issue in the relatively poor performance of the used prediction services. Since the *P.falciparum* proteins are likely to be differently distributed than the proteins used to train the prediction services, these services perform poorly when applied to our test set. This result supports our claim that providing curated datasets with high-quality labels for model training is essential for maximising the potential of computational prediction methods on biological prediction tasks like the pre-screening of *P.falciparum* protein antigen candidates. Therefore, our proposed *PlasmoFAB* benchmark offers a solution to one fundamental obstacle in utilizing computational prediction methods in the development process of drugs and vaccines against malaria.

One goal of developing *PlasmoFAB* was to provide the malaria research community with a tool to utilize machine learning in protein antigen exploration processes. However, the potential target user group of *PlasmoFAB* can only benefit from the data if it fulfils two basic requirements. First, potential users have to be enabled to reliably find, access, and reuse data. And second, potential users have to be able to make an informed decision whether the data are applicable for their specific problem. We tackle the first problem by making *PlasmoFAB* publicly available via Zenodo, which is a platform by researchers for researchers that aims to support open science. By uploading our dataset to Zenodo, we ensure that the FAIR principles (Wilkinson et al. 2016) are taken into account. Additionally, we release *PlasmoFAB* in form of comma-separated values (CSV) files. This file format is universally used in different research communities and should maximize the number of researchers that can use our dataset. Furthermore, we created a datasheet for *PlasmoFAB* as described in Gebru et al. (2021). With this datasheet, we provide information about the motivation behind creating *PlasmoFAB*, the creation process, the assumptions made, and applicable use cases. Users who are interested in using *PlasmoFAB* can use the datasheet to make an informed decision about the applicability.

## 5 Conclusion

With this work, we introduce *PlasmoFAB*, a new and carefully curated benchmark for the training of models for *P.falciparum* protein antigen candidate prediction. The benchmark was created by manually validating extracellular, surface-exposed, and intracellular *P.falciparum* proteins to ensure high-quality labels for every sample in the dataset. Such a curated benchmark is an important prerequisite to incorporate learning models into pre-screening protocols for protein antigen candidates.

We furthermore compared commonly used prediction models with publicly available prediction services on the *P.falciparum* protein antigen candidate prediction task. Our results show the limitations of existing prediction services, which are vastly outperformed by simpler prediction models that are specifically trained for *P.falciparum* protein antigen candidate prediction.

We are confident that our contribution provides a tool that can be used to help the research community to explore the vast number of *P.falciparum* proteins with unknown functionality and identify new targets for drugs and vaccines against malaria.
